# NMR pseudocontact shifts in a symmetric protein homotrimer

**DOI:** 10.1007/s10858-020-00329-7

**Published:** 2020-07-03

**Authors:** Thomas Müntener, Raphael Böhm, Kenneth Atz, Daniel Häussinger, Sebastian Hiller

**Affiliations:** 1 Biozentrum, University of Basel, Klingelbergstrasse 70, 4056 Basel, Switzerland; 2 Department of Chemistry, University of Basel, St. Johanns-Ring 19, 4056 Basel, Switzerland

**Keywords:** Solution NMR spectroscopy, Pseudocontact shift, Paramagnetism, Lanthanide chelating tag, Homotrimer, Chaperone Skp

## Abstract

NMR pseudocontact shifts are a valuable tool for structural and functional studies of proteins. Protein multimers mediate key functional roles in biology, but methods for their study by pseudocontact shifts are so far not available. Paramagnetic tags attached to identical subunits in multimeric proteins cause a combined pseudocontact shift that cannot be described by the standard single-point model. Here, we report pseudocontact shifts generated simultaneously by three paramagnetic Tm-M7PyThiazole-DOTA tags to the trimeric molecular chaperone Skp and provide an approach for the analysis of this and related symmetric systems. The pseudocontact shifts were described by a “three-point” model, in which positions and parameters of the three paramagnetic tags were fitted. A good correlation between experimental data and predicted values was found, validating the approach. The study establishes that pseudocontact shifts can readily be applied to multimeric proteins, offering new perspectives for studies of large protein complexes by paramagnetic NMR spectroscopy.

**Electronic supplementary material:**

The online version of this article (10.1007/s10858-020-00329-7) contains supplementary material, which is available to authorized users.

## Introduction

Precise descriptions of protein structure and dynamics are key to understand biological functionality. NMR spectroscopy has proven a valuable source to gain such information under native or native-like conditions for highly relevant systems. Thereby, different geometrical restraints can be used to determine the structure of a protein. While residual dipolar couplings (RDCs) determine relative orientations of bond vectors (Chen and Tjandra 2012), nuclear Overhauser effects (NOEs) provide short-range distance information (Wagner and Wüthrich 1982; Williamson 2009). On the other hand, the two paramagnetic effects paramagnetic relaxation enhancement (PRE) and pseudocontact shift (PCS), which can be introduced into proteins by various lanthanide-chelating tags (LCTs), have proven to provide valuable distance- and orientation-dependent information over extremely long distances exceeding 100 Å (Joss and Häussinger 2019a; Nitsche and Otting 2017; Wang et al. 2007; Bertini et al. 2002a,b; Gochin and Roder 1995; Pearce et al. 2017). LCTs based on the DOTA (1,4,7,10-tetraazacyclododecane-1,4,7,10-tetraacetic acid) framework provide large PCSs and are usually attached to the protein surface via one or two cysteine residues (Joss and Häussinger 2019a; Nitsche and Otting 2017; Keizers et al. 2007; Prudencio et al. 2004). Recent developments of LCTs with reduction-stable linkages have enabled studies under physiologically relevant conditions in living cells (Müntener et al. 2016, 2018; Pan et al. 2016; Joss and Häussinger 2019b; Liu et al. 2014; Hikone et al. 2016). Nevertheless, many soluble proteins, as well as membrane proteins, form homomultimeric complexes (Hashimoto et al. 2011; Ali and Imperiali 2005; Goodsell and Olson 2000), which are not readily accessible by PCS NMR spectroscopy due to formation of multi-tagged protein complexes. Here, we report a generalized approach to study pseudocontact shift effects generated by multiple paramagnetic centers and demonstrate this approach on the homotrimeric protein Skp (Walton and Sousa 2004; Korndörfer et al. 2004; Burmann et al. 2013; Callon et al. 2014).

## Methods and materials

### Cloning and mutagenesis of Skp single-cysteine mutants

The original Skp gene without its signal sequence was cloned from genomic DNA through NdeI and XhoI into the pET28b expression vector (Novagen) containing a thrombin-cleavable N-terminal His_6_-tag (Burmann et al. 2013). The QuikChange II mutagenesis protocol (Stratagene) was used to introduce the mutation S126C. PCR primers were obtained from Microsynth.

### Expression, purification of Skp (S126C)

Uniformly [^2^H,^15^N]-Skp(S126C) was produced as previously described (Burmann et al. 2013). In brief, BL21(DE3)-LEMO cells (NEB) were transformed with the plasmid and grown at 37 °C in D_2_O-based M9 medium, with 1 g ^15^N-ammonium chloride, containing 30 mg/mL kanamycin to OD_600_ = 0.6 and then for an additional 30 min at 20 °C before expression was induced with 0.4 mM IPTG. Cells were harvested 18 h after induction, resuspended in lysis buffer (25 mM HEPES, pH 7.5, 300 mM NaCl, 10 mM imidazole, DNase (0.01 mg/mL), RNase (0.02 mg/mL) and inhibitor cocktail (cOmplete EDTA-free protease inhibitor, Roche) at a 4:1 buffer/pellet weight ratio and lysed by sonication. The soluble lysate was separated from cell debris and other components by centrifugation at 14′000×*g* for 60 min at 4 °C and then applied to Ni^2+^ beads (Genscript) equilibrated with lysis buffer. The wash buffer contained 30 mM imidazole and the elution buffer 500 mM imidazole. Skp(S126C) elution fractions were dialyzed against 25 mM HEPES, pH 7.5, 300 mM NaCl overnight at 4 °C to remove imidazole. The dialyzed Skp sample was denatured with 6 M guanidinium hydrochloride, applied to Ni^2+^ beads, and eluted with 500 mM imidazole. 10 mM DTT were added to the Skp elution fraction before dialysis against lysis buffer containing 5 mM DTT over night at 4 °C. As a final purification step, Skp(S126C) was buffer exchanged to NMR buffer (20 mM MES, pH 6.5, 150 mM NaCl and 1 mM DTT) using size exclusion chromatography (Superdex200 16/600). Refolded Skp(S126C) eluted from a size exclusion column (HiLoad 16/600 Superdex 200 pg) at an elution volume of 79 mL, which corresponds to trimeric wild-type Skp and is substantially different from monomeric wild-type Skp. A 2D [^15^N,^1^H]-TROSY spectrum was recorded to ensure that the S126C mutation had no effect on overall Skp structure. Just as for wild-type Skp (Burmann et al. 2013), the spectrum of the Skp(S126C) mutant features a single resonance for each amide moiety, resulting from superimposition of the individual signals from the three promoters due to the molecular symmetry. Afterwards, Skp was either directly used for site-specific spin labeling or stored at − 20 °C until use.

### Site-specific spin labeling of Skp(S126C)

Spin labeling with the lanthanide chelating tag, Tm-M7PyThiazol-DOTA, of the introduced cysteine S126C was done according to published protocols (Müntener et al. 2018). In brief, protein solution (100 μM Skp trimer) in NMR buffer was exchanged to tagging buffer (50 mM sodium phosphate, pH 7, 150 mM NaCl, 0.3 mM TCEP) in Vivaspin 5-kDa concentrators (Vivascience). A sevenfold excess of Tm-M7PyThiazol-DOTA dissolved in tagging buffer was added to the protein solution, and this was followed by incubation overnight at room temperature under shaking at 300 rpm. To remove unreacted Tm-M7PyThiazol-DOTA, the buffer was exchanged back to NMR buffer using 5-kDa concentrators.

### NMR measurements of Skp(S126C)

All NMR spectra were recorded at 25 °C on a Bruker Avance-700 spectrometer equipped with a cryogenic triple-resonance probe. The sample contained 0.3–0.4 mM [^2^H,^15^N]-labeled protein Skp in 20 mM MES pH 6.5 with 150 mM NaCl, 1 mM DTT and 5%/95% D_2_O/H_2_O. The proton chemical shifts were referenced to internal DSS and those for carbon-13 and nitrogen-15 were indirectly referenced. The 2D [^15^N,^1^H]-TROSY and semi-TROSY were recorded in a total experiment time of 14 h. The ^1^H carrier was centered on the water resonance, the ^15^N carrier at 118 ppm. The interscan delay was set to 1 s. In the direct dimension, 1024 complex points were recorded in an acquisition time of 90 ms, multiplied with a 90°-shifted sine bell, zero-filled to 2048 points and Fourier transformed. In the indirect dimension, 200 complex points were measured with a maximal evolution time of 93 ms, multiplied with a 90°-shifted sine bell, zero-filled to 512 points and Fourier transformed. Polynomial baseline corrections were applied in all dimensions.

### PCS tensor fitting

Paramagnetic anisotropic susceptibility tensors were fitted using an in-house Python script. The source code is available upon request.

## Results and discussion

The PCS of a nuclear spin is the difference in chemical shift between the paramagnetic sample and a diamagnetic reference. The shift arises from dipolar through-space interaction between the nuclear spin and a paramagnetic center that has an anisotropic electron g-factor. Using the point–dipole approximation, the PCS caused by a single paramagnetic center can be described by the following expression (Bertini et al. 2002a):

$$ \sigma_{\text{PCS}} = \frac{1}{{12{\uppi }}}\left[ {\Delta {\upchi}_{\text{ax}} \frac{{2z^{2} - x^{2} - y^{2} }}{{r^{5} }} + \frac{3}{2}\Delta {\upchi }_{\text{rh}} \frac{{x^{2} - y^{2} }}{{r^{5} }}} \right] $$

where Δχ_ax_ and Δχ_rh_ are the axial and rhombic components of the anisotropy of the magnetic susceptibility tensor χ, respectively, *r* is the distance from the nucleus to the paramagnetic center and *x*, *y*, *z* describe the Cartesian coordinates of the nucleus in the tensor frame. The tensor frame has the paramagnetic center at the origin and the axes oriented according to the symmetry of the PCS.

For a given three-dimensional protein structure and experimental pseudocontact shifts, the anisotropic part of the magnetic susceptibility tensor can be determined from a sufficiently large data set by fitting eight free parameters (Schmitz et al. 2008). These are Δχ_ax_ and Δχ_rh_, which describe the shape and size of the tensor, *x*_metal_, *y*_metal_ and *z*_metal_, which define the position of the paramagnetic center in the molecular frame, and the three Euler angles *α*, *β* and *γ*, which define the relative orientation of the tensor frame with respect to the molecular frame.

**Fig. 1:**
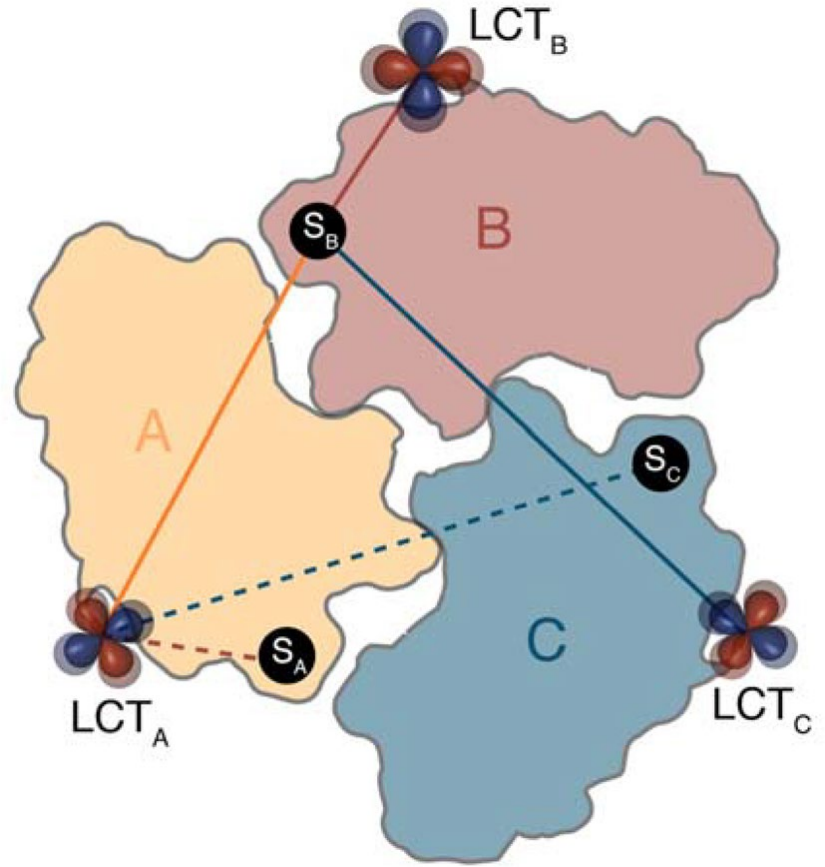
Calculation of pseudocontact shifts in a symmetric homotrimer. The three symmetrically attached LCTs are labelled LCT_A–C_. In a C_3_ symmetric arrangement, the PCS effect experienced by spin S_B_ generated by all LCTs is identical to the sum of all effects generated by a single LCT (e.g. LCT_A_, dashed lines) on the symmetry equivalent spins S_A_, S_B_ and S_C_

For a system with multiple paramagnetic centers, the observed PCS is the sum of the individual PCS contributions generated by each paramagnetic center (Velyvis et al. 2009). The tensor frames of these individual centers are in general not aligned with respect to each other, resulting in the following expression:

$$ \sigma_{\text{PCS}} = \mathop \sum \limits_{i = 1}^{n} \frac{1}{{12{\uppi }}}\left[ {\Delta {\upchi}_{{\text{ax}}_{i}} \frac{{2z_{i}^{2} - x_{i}^{2} - y_{i}^{2}}}{r_{i}^{5} } + \frac{3}{2}\Delta {\upchi}_{{\text{rh}}_{i}} \frac{{x_{i}^{2} - y_{i}^{2} }}{r_{i}^{5}}} \right] $$

where *i* indexes the individual paramagnetic tensors. In principle, this equation requires fitting of eight parameters for each paramagnetic center. Importantly, however, for symmetric systems the number of free parameters reduces substantially. For example, in the case of a homotrimeric protein with C_3_ symmetry, the three metal positions and the corresponding Euler angles are related by 120° and 240° rotations around the symmetry axis (Fig. 1). Furthermore, each of the three LCTs has identical Δχ_ax_ and Δχ_rh_ values.

Thus, a significant simplification can be achieved by realizing that the PCS generated by all three LCTs on a specific spin is the sum of all PCSs generated by one LCT on all three symmetry equivalent spins in chains A, B and C (Fig. 1). This property allows for a direct fitting procedure as only one set of eight free parameters describing one paramagnetic center needs to be calculated. Practically, the components of the anisotropy of the magnetic susceptibility tensor, the Euler angles, and one metal position can be fitted by minimizing a quadratic cost function *f* using Quasi-Newton methods implemented in the Python library SciPy:

$$ f = \mathop \sum \limits_{j} \left[ {PCS_{j}^{exp} - \left( {PCS_{{j_{A} }}^{calc} + PCS_{{j_{B} }}^{calc} + PCS_{{j_{C} }}^{calc} } \right)} \right]^{2} $$

where *j* defines the nuclear spins and *A*, *B* and *C* denote the corresponding C_3_-symmetric chains. Notably, in a C_3_-symmetric environment, three different local minima of *f* exist, each of which corresponds to the tag positioned on one of the three chains A, B or C. Changing the initial starting position of the iteration close to one of the chains provides access to all three possible solutions in three successive minimizations with the same input data.

**Fig. 2:**
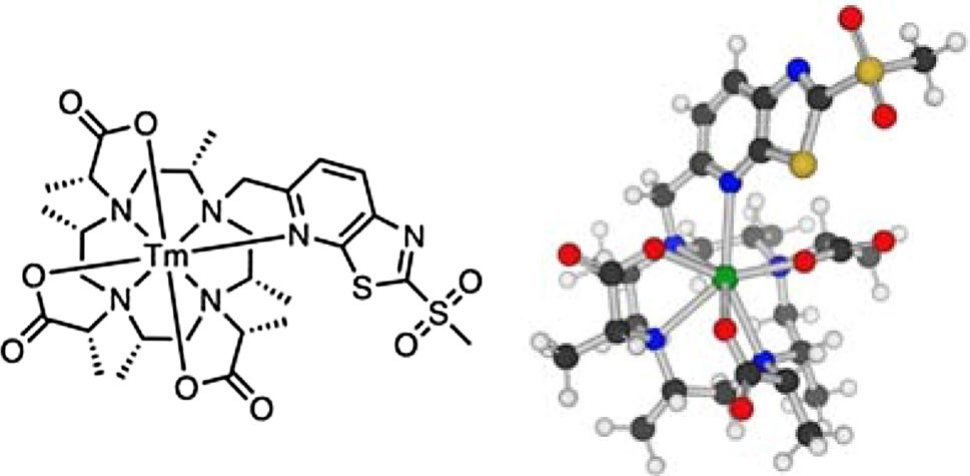
2D representation and 3D Structure of Tm-M7PyThiazol-SO_2_-Me-DOTA

**Fig. 3:**
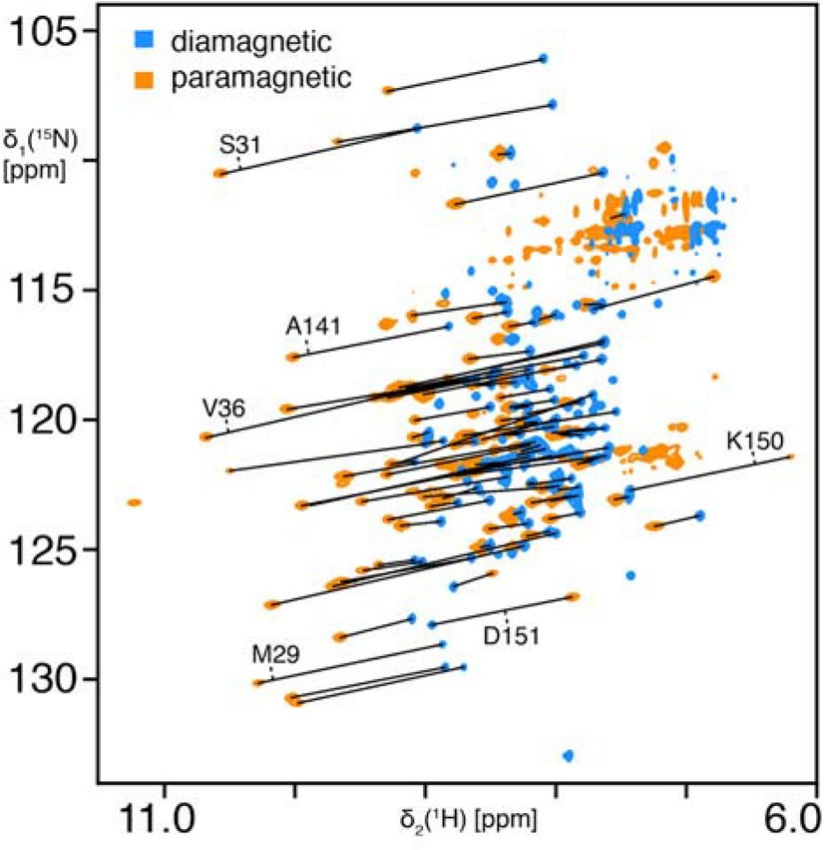
NMR pseudocontact shifts in the homotrimeric protein Skp. Superposition of 2D [^15^N,^1^H]-TROSY spectra of Skp(S126C) (diamagnetic, blue) and coupled to paramagnetic Tm-M7Py-Thiazol-DOTA (orange). Measured pseudocontact shifts are illustrated with a black line and a few selected residues are labelled with their assignment. Spectra were recorded in aqueous buffer (25 mM MES, 150 mM NaCl, pH 6.5) at 700 MHz Larmor frequency and 25 °C

We prepared uniformly labelled [^2^H,^15^N]-Skp with a single cysteine introduced at position 126 (Skp(S126C)), following established protocols (Burmann et al. 2013). The cysteine mutation was specifically introduced into helix 5, which is a stable structural element of Skp, providing good solvent accessibility and limited molecular motion. First, we prepared a diamagnetic reference sample using untagged [^2^H,^15^N]-Skp(S126C). Second, we prepared a paramagnetically tagged [^2^H,^15^N]-Skp(S126C) sample, using Tm-M7PyThiazole-SO_2_-Me-DOTA. Thereby, the metal Tm was chosen, because it is among the three lanthanides showing the strongest shift (Tm, Dy, Tb). Furthermore, in the M7PyThiazole-SO_2_-Me-DOTA tag, Tm has been shown to produce PCSs at more distant residues due to its more favorable orientation of the tensor isosurfaces, while exhibiting smaller PREs compared to Dy (Müntener et al. 2018). Tagging was performed overnight at room temperature using a sevenfold excess of the paramagnetic spin label at pH 7.0 under reducing conditions. We recorded a 2D [^15^N,^1^H]-TROSY at 700 MHz Larmor frequency and 25 °C. For the non-shifted, diamagnetic reference spectrum, we observed only one single, coherent set of resonances and no significant chemical shift changes, in good agreement with previously reported data (Burmann et al. 2013). The paramagnetically shifted spectrum also exhibited one single and coherent set of resonances, indicating a highly selective and complete threefold tagging of Skp(S126C) by Tm-M7PyThiazole-SO_2_-Me-DOTA (Fig. 2). The observed PCSs were mostly positive, reaching up to 5 ppm (Fig. 3). Notably, an incomplete tagging or disruption of the protein structure would break the C_3_ symmetry, leading to an asymmetric structure and resulting in tripling of the observed resonances (Gaponenko et al. 2002).

For further analysis of the observed PCSs, the aforementioned three-positions-one-tensor method was used. While the available crystal structures of Skp (PDB 1U2M, 1SG2) report only partly resolved polypeptide chains, the protein is known to exhibit fast dynamics in aqueous solution, leading to a complete symmetry equivalence of the three subunits on the NMR timescale (Burmann et al. 2013). This property allowed us to extend the incomplete regions of the crystal structure 1U2M by duplicating the complete chain A and superimposing it on the incomplete parts of chain B and C. The modified structure features a complete set of atomic coordinates for all residues in all chains, and exhibits a slight asymmetry due to crystal contacts. Assignment of the diamagnetic reference spectrum was taken from the BMRB entry 19407 and the paramagnetic spectrum was assigned in an iterative fashion. An initial set of around ten unambiguous pseudocontact shifts were used to approximate the tensor. Back-prediction using this initial tensor values and the sequence-specific resonance assignment in the diamagnetic reference spectrum provided access to further assignments of the paramagnetic spectrum, which were used to refine the tensor. This procedure was repeated iteratively until no further assignments were possible. The tensor parameters converged with the input data from around 40 PCSs and stayed stable upon inclusion of the data from all other residues.

**Fig. 4:**
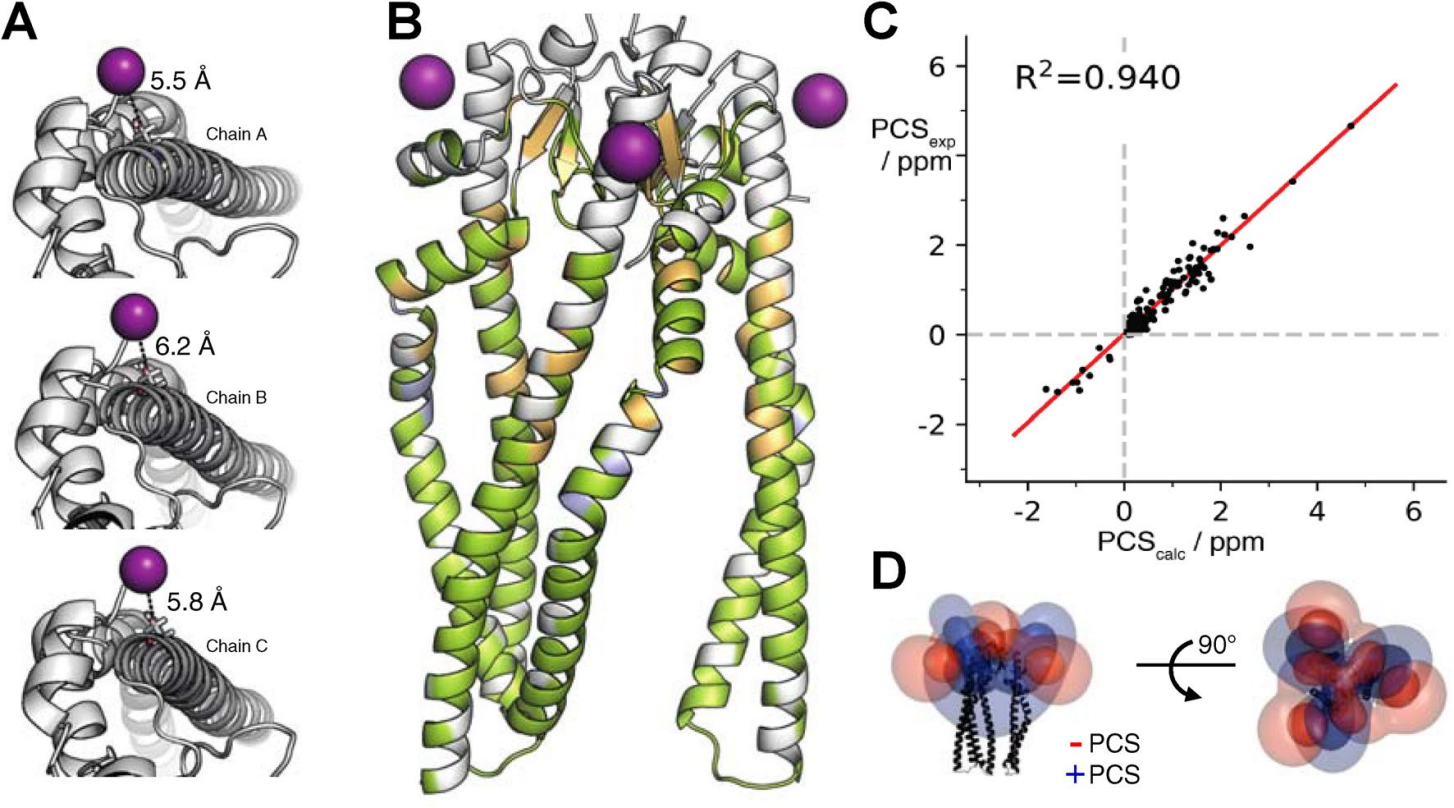
NMR pseudocontact shifts in Skp(S126C). **a** Location of the three fitted LCT positions (purple spheres) for chains A–C. The distance to the corresponding Cys C^β^ is indicated by a dashed line. **b** Display of PCS deviations on the structure of Skp. Residues with a total PCS deviation of less than 0.2 ppm are colored in green. Residues in orange or blue have a deviation of larger 0.2 ppm or 0.4 ppm, respectively. Unassigned residues are colored in gray. The metal positions of the LCTs are indicated as purple spheres. **c** Correlation graph between PCSs back calculated from the modified X-ray structure and the experimental PCS tabulated in Table S1. **d** Isosurfaces generated by three Tm loaded M7PyThiazol-DOTA tags coupled to Skp(S126C). Red isosurfaces indicate a shift of − 1.0/ − 0.2 ppm (inner/outer surface). Blue isosurfaces indicate a corresponding positive shift

Three different sets of tensor parameters were fitted using different starting positions for the metal corresponding to the three attachment sites of the spin-label (Table 1). The obtained tensor parameters are remarkably similar and show only small deviations, which are well explained by the lack of a perfect C_3_ symmetry in the crystal structure. As can be seen from the almost identical tensor parameters, the three metals form a near-perfect equilateral triangle with average distances of 5.5–6.2 Å to the corresponding C^α^ atom of cysteine C126 (Fig. 4a).

**Table 1:** PCS tensor parameters of trimeric Skp(S126C) with three Tm-M7PyThiazol-DOTA tags

	Chain A	Chain B	Chain C
Δχ_ax_ (10^–32^ m^3^)	− 43.0	− 42.2	− 43.0
Δχ_rh_ (10^–32^ m^3^)	− 7.8	− 8.5	− 8.5
r_metal_ (Å)^a,c^	25.1	25.5	25.6
θ_metal_ (°)^a,c^	241.0	0.0	121.0
α (°)^b,c^	96.0	93.9	91.0
β (°)^b,c^	120.3	63.01	63.5
γ (°)^b,c^	71.7	132.3	6.5

^a^Metal position given in polar coordinates

^b^Euler angles given in ‘ZYZ’ convention

^c^Coordinates have been rotated such that the origin (0,0,0) is in the center of gravity of the three metals and all metals lie in the xy-plane

Paramagnetic alignment induced sizeable RDCs reaching absolute magnitudes of up to 40 Hz at 700 MHz Larmor frequency (SI Table 2) and in addition, residual anisotropic chemical shifts (RACS) are to be expected. We analyzed the effect of partial alignment on the quality of the fit and the tensor parameters by using only proton PCS for which the errors on our observed PCS can be neglected as both contributions from RACS (John et al. 2005) and RDCs are small. We found only marginal changes in the quality of the fit and the tensor parameters and as a consequence all measured PCS were used.

Upon close inspection we found that experimental PCSs are in good agreement with the back-calculated values. Larger deviations occurred in regions previously identified to undergo large backbone motions (Fig. 4b, c) (Burmann et al. 2013). A number of resonances in close proximity to the tagging sites are line-broadened beyond the detection limit due to the PRE effect (Figure S3). The three LCTs generate overlapping isosurfaces resulting in a propeller-like shape and a significantly larger positive isosurface covering a large portion of the arms of Skp (Fig. 4d). These extended isosurfaces allow in favorable cases the study of structural changes in remote distances greater than 100 Å away from the tagging site.

## Conclusion

In conclusion, we derived a generalised approach for the interpretation and analysis of PCS generated by multiple symmetric paramagnetic centers. We also established a substantially simplified procedure for fitting of the tensor parameters, which is readily extendable to any C_n_ symmetric system. On the homotrimeric protein Skp we demonstrated the feasibility of measuring PCS with three attached LCTs. The applied model resulted in good agreement between experimental and back-calculated PCS, showing larger deviations in highly dynamic regions. We envision that the irreversible linkage between the protein and the LCT in combination with the method reported herein will allow the study of large biologically relevant homomultimeric systems under physiological conditions, providing valuable insights into their long-range structure, interactions and dynamics.

## Electronic supplementary material

Below is the link to the electronic supplementary material.

Supplementary file1 (DOCX 370 kb)

## Data Availability

The Supporting Information is available free of charge on the Publications website.

## Code availability

Python source code is available upon request.
