# Supplementary material for: NMR pseudocontact shifts in a symmetric protein homotrimer
Source: J Biomol NMR. 2020 Jul 3;74(8):413–9. doi: 10.1007/s10858-020-00329-7 (PMC7508745; doi:10.1007/s10858-020-00329-7)
Supplement: Supplementary file 1 — Supplementary file1 (DOCX 370 kb) [file 10858_2020_329_MOESM1_ESM.docx]

Supplementary information

NMR pseudocontact shifts in a symmetric protein homotrimer

Thomas Müntener, Raphael Böhm, Kenneth Atz, Daniel Häussinger, Sebastian Hiller^*^

* corresponding author: sebastian.hiller@unibas.ch

This file contains:

- Supplementary Tables S1 & S2
- Supplementary Figure S3

Table S1. Chemical shift assignment for diamagnetic Skp(S126C), paramagnetic Skp(S126C) tagged with Tm-M7PyThiazol-DOTA and the resulting PCSs.

|  | diamagnetic Skp(S126C) | | paramagnetic Skp(S126C) | | PCS | |
| --- | --- | --- | --- | --- | --- | --- |
|  | δ(^1^H^N^) [ppm] | δ (^15^N) [ppm] | δ (^1^H^N^) [ppm] | δ (^15^N) [ppm] | PCS(^1^H^N^) [ppm] | PCS(^15^N) [ppm] |
| 11Ser | 7.857 | 114.36 | - | - | - | - |
| 25Ala | 7.713 | 115.73 | 6.794 | 114.49 | -0.919 | -1.25 |
| 26Ile | 8.784 | 126.42 | 8.486 | 125.91 | -0.298 | -0.50 |
| 27Val | 8.848 | 115.15 | - | - | - | - |
| 28Asn | 8.203 | 118.06 | 9.286 | 119.15 | 1.083 | 1.09 |
| 29Met | 8.875 | 128.66 | 10.281 | 130.14 | 1.406 | 1.47 |
| 30Gly | 8.004 | 124.37 | 9.707 | 126.41 | 1.703 | 2.04 |
| 31Ser | 9.065 | 108.80 | 10.568 | 110.53 | 1.503 | 1.73 |
| 32Leu | 7.791 | 117.54 | 9.145 | 118.77 | 1.354 | 1.23 |
| 33Phe | 8.038 | 121.39 | 9.948 | 123.31 | 1.910 | 1.92 |
| 34Gln | 8.618 | 118.42 | 10.058 | 119.59 | 1.440 | 1.17 |
| 35Gln | 7.636 | 117.06 | 9.202 | 118.75 | 1.566 | 1.69 |
| 36Val | 8.449 | 118.08 | 10.681 | 120.69 | 2.232 | 2.60 |
| 37Ala | 9.000 | 120.93 | 10.494 | 121.96 | 1.494 | 1.03 |
| 38Gln | 8.372 | 122.02 | 9.487 | 123.15 | 1.115 | 1.13 |
| 39Lys | 7.651 | 117.68 | 9.014 | 119.02 | 1.363 | 1.34 |
| 40Thr | 8.160 | 120.85 | 9.622 | 122.17 | 1.462 | 1.32 |
| 41Gly | 8.029 | 107.87 | 9.672 | 109.29 | 1.643 | 1.42 |
| 42Val | 7.642 | 110.49 | 8.770 | 111.69 | 1.128 | 1.20 |
| 43Ser | 7.598 | 121.07 | 8.642 | 121.94 | 1.044 | 0.86 |
| 46Leu | 8.307 | 118.54 | 8.648 | 118.66 | 0.341 | 0.12 |
| 47Glu | 8.019 | 122.58 | 9.011 | 122.96 | 0.992 | 0.38 |
| 48Asn | 8.217 | 119.90 | 8.422 | 120.03 | 0.205 | 0.13 |
| 49Glu | 8.200 | 117.36 | 8.666 | 117.65 | 0.466 | 0.29 |
| 50Phe | 8.507 | 123.10 | 9.283 | 123.84 | 0.776 | 0.74 |
| 51Lys | 7.735 | 120.40 | 8.029 | 120.60 | 0.294 | 0.20 |
| 52Gly | 8.149 | 120.08 | 8.243 | 120.13 | 0.094 | 0.05 |
| 53Arg | 8.098 | 106.11 | 9.291 | 107.34 | 1.193 | 1.23 |
| 54Ala | 8.211 | 124.00 | 8.510 | 124.20 | 0.299 | 0.20 |
| 55Ser | 8.880 | 123.91 | 9.188 | 124.08 | 0.308 | 0.16 |
| 56Glu | 8.372 | 115.84 | 8.633 | 116.09 | 0.261 | 0.25 |
| 57Leu | 7.815 | 123.59 | 8.051 | 123.81 | 0.236 | 0.22 |
| 58Gln | 7.982 | 122.54 | 8.110 | 122.65 | 0.128 | 0.11 |
| 59Arg | 8.228 | 119.45 | 8.296 | 119.49 | 0.068 | 0.04 |
| 60Met | 7.833 | 123.27 | - | - | - | - |
| 62Thr | 8.395 | 121.77 | 8.569 | 121.87 | 0.174 | 0.10 |
| 63Asp | 8.163 | 116.24 | 8.356 | 116.40 | 0.193 | 0.16 |
| 64Leu | 8.051 | 124.29 | 8.223 | 124.48 | 0.172 | 0.18 |
| 65Gln | 8.601 | 122.62 | 8.863 | 123.02 | 0.262 | 0.40 |
| 66Ala | 8.437 | 121.64 | 8.652 | 121.72 | 0.215 | 0.07 |
| 67Lys | 9.086 | 125.40 | 9.360 | 125.60 | 0.274 | 0.20 |
| 68Met | 8.444 | 125.20 | - | - | - | - |
| 69Lys | 8.127 | 120.99 | 8.264 | 121.21 | 0.137 | 0.23 |
| 70Lys | 8.496 | 118.67 | 8.606 | 118.66 | 0.110 | -0.01 |
| 71Leu | 7.888 | 120.51 | 7.983 | 120.53 | 0.095 | 0.02 |
| 72Gln | 8.275 | 120.54 | 8.368 | 120.57 | 0.093 | 0.03 |
| 73Ser | 7.651 | 115.55 | 7.778 | 115.55 | 0.127 | 0.00 |
| 74Met | 7.471 | 112.07 | 7.575 | 112.22 | 0.104 | 0.15 |
| 75Lys | 7.444 | 122.97 | 7.550 | 123.07 | 0.106 | 0.10 |
| 76Ala | 8.504 | 124.83 | 8.577 | 124.91 | 0.073 | 0.07 |
| 77Gly | 8.265 | 123.56 | 8.347 | 123.68 | 0.082 | 0.12 |
| 78Ser | 8.348 | 109.74 | 8.441 | 109.77 | 0.093 | 0.03 |
| 81Thr | 7.895 | 121.17 | - | - | - | - |
| 82Lys | 8.003 | 115.95 | 8.125 | 116.13 | 0.122 | 0.19 |
| 83Leu | 8.321 | 121.40 | - | - | - | - |
| 84Glu | 7.805 | 120.58 | 7.960 | 120.59 | 0.155 | 0.01 |
| 85Lys | 7.908 | 119.52 | - | - | - | - |
| 86Asp | 7.676 | 121.47 | 7.804 | 121.64 | 0.128 | 0.17 |
| 87Val | 8.181 | 122.41 | 8.215 | 122.49 | 0.034 | 0.07 |
| 88Met | 8.863 | 120.81 | 9.003 | 120.90 | 0.140 | 0.09 |
| 89Ala | 8.211 | 119.48 | 8.317 | 119.55 | 0.106 | 0.07 |
| 90Gln | 7.831 | 122.92 | 8.035 | 123.12 | 0.204 | 0.21 |
| 91Arg | 7.846 | 117.92 | 8.087 | 118.09 | 0.241 | 0.17 |
| 92Gln | 8.084 | 121.13 | 8.291 | 121.36 | 0.207 | 0.23 |
| 93Thr | 7.722 | 120.32 | 7.856 | 120.40 | 0.134 | 0.08 |
| 94Phe | 8.216 | 118.33 | 8.404 | 118.48 | 0.188 | 0.16 |
| 95Ala | 8.753 | 123.18 | 8.962 | 123.33 | 0.209 | 0.15 |
| 96Gln | 8.238 | 120.99 | 8.297 | 121.07 | 0.059 | 0.08 |
| 97Lys | 8.620 | 120.83 | 8.784 | 120.96 | 0.164 | 0.13 |
| 98Ala | 9.085 | 121.60 | 9.265 | 121.70 | 0.180 | 0.10 |
| 99Gln | 7.826 | 122.60 | 7.980 | 122.69 | 0.154 | 0.10 |
| 100Ala | 8.017 | 120.27 | 8.406 | 120.58 | 0.389 | 0.31 |
| 101Phe | 7.885 | 122.94 | 8.186 | 123.19 | 0.301 | 0.25 |
| 102Glu | 8.012 | 119.24 | 8.353 | 119.51 | 0.341 | 0.26 |
| 103Gln | 8.000 | 119.98 | 8.563 | 120.76 | 0.563 | 0.77 |
| 104Asp | 7.809 | 119.30 | 8.212 | 119.77 | 0.403 | 0.46 |
| 105Arg | 7.723 | 121.59 | 7.832 | 121.69 | 0.109 | 0.10 |
| 106Ala | 8.976 | 120.48 | 9.097 | 120.67 | 0.121 | 0.19 |
| 107Arg | 7.883 | 122.26 | 8.193 | 122.50 | 0.310 | 0.25 |
| 109Ser | 8.506 | 119.51 | 9.082 | 120.02 | 0.576 | 0.51 |
| 110Asn | 8.381 | 115.46 | 9.103 | 115.97 | 0.722 | 0.51 |
| 112Glu | 8.048 | 118.81 | 8.428 | 119.14 | 0.380 | 0.33 |
| 113Arg | 8.489 | 118.31 | 9.397 | 119.15 | 0.908 | 0.84 |
| 114Gly | 7.614 | 121.39 | 8.761 | 122.15 | 1.147 | 0.76 |
| 115Lys | 8.489 | 110.85 | - | - | - | - |
| 116Leu | 7.848 | 114.96 | 9.101 | 116.18 | 1.253 | 1.22 |
| 117Val | 8.203 | 121.41 | 9.877 | 123.29 | 1.674 | 1.88 |
| 118Thr | 8.243 | 124.86 | 10.178 | 127.13 | 1.935 | 2.28 |
| 119Arg | 8.342 | 116.92 | 10.524 | 118.88 | 2.182 | 1.96 |
| 120Ile | 7.831 | 121.35 | - | - | - | - |
| 121Gln | 8.379 | 121.55 | - | - | - | - |
| 124Val | 7.429 | 126.00 | - | - | - | - |
| 125Lys | 8.613 | 119.10 | - | - | - | - |
| 127Val | 7.700 | 119.36 | - | - | - | - |
| 128Ala | 7.981 | 122.13 | - | - | - | - |
| 129Asn | 8.678 | 122.37 | - | - | - | - |
| 131Gln | 7.842 | 115.59 | - | - | - | - |
| 135Leu | 8.336 | 125.15 | - | - | - | - |
| 136Val | 7.627 | 120.33 | 7.916 | 120.43 | 0.289 | 0.11 |
| 137Val | 9.026 | 125.47 | 9.473 | 125.80 | 0.447 | 0.33 |
| 138Asp | 9.105 | 127.67 | 9.652 | 128.39 | 0.547 | 0.72 |
| 139Ala | 8.648 | 125.33 | 9.644 | 126.23 | 0.996 | 0.91 |
| 140Asn | 8.851 | 129.54 | 10.025 | 130.70 | 1.174 | 1.16 |
| 141Ala | 8.826 | 116.41 | 10.012 | 117.59 | 1.186 | 1.17 |
| 142Val | 8.197 | 120.98 | 9.299 | 122.11 | 1.102 | 1.14 |
| 143Ala | 7.543 | 119.69 | 8.709 | 120.61 | 1.166 | 0.92 |
| 144Tyr | 8.710 | 129.53 | 9.976 | 130.91 | 1.266 | 1.38 |
| 145Asn | 7.628 | 116.93 | 8.842 | 118.38 | 1.214 | 1.45 |
| 146Ser | 6.897 | 123.69 | 7.242 | 124.10 | 0.345 | 0.41 |
| 147Ser | 8.894 | 119.39 | - | - | - | - |
| 148Asp | 8.653 | 114.29 | - | - | - | - |
| 149Val | 7.716 | 119.64 | - | - | - | - |
| 150Lys | 7.433 | 122.71 | 6.211 | 121.44 | -1.222 | -1.28 |
| 151Asp | 8.952 | 127.90 | 7.878 | 126.83 | -1.074 | -1.07 |
| 155Asp | 8.723 | 121.96 | 7.934 | 121.40 | -0.789 | -0.56 |
| 156Val | 7.719 | 119.03 | 9.213 | 121.68 | 1.494 | 2.65 |
| 157Leu | 8.410 | 120.77 | 11.831 | 125.43 | 3.421 | 4.66 |
| 158Lys | 8.071 | 120.30 | - | - | - | - |
| 159Gln | 7.221 | 115.53 | - | - | - | - |
| 160Val | 7.496 | 115.95 | - | - | - | - |
| 161Lys | 7.334 | 121.18 | - | - | - | - |

Table S2. Residual dipolar couplings of Skp(S126C) tagged with Tm-M7PyThiazol-DOTA at 700 MHz.

| Residue number | Nucleus 1 | Nucleus 2 | RDC [Hz] |
| --- | --- | --- | --- |
| 25 | N | H | 2.5 |
| 28 | N | H | 19.4 |
| 30 | N | H | 22.6 |
| 31 | N | H | 8.5 |
| 33 | N | H | 10.9 |
| 34 | N | H | 0.9 |
| 35 | N | H | 4.8 |
| 36 | N | H | 21.1 |
| 37 | N | H | -34.2 |
| 38 | N | H | -39.3 |
| 39 | N | H | 2.9 |
| 40 | N | H | 6.1 |
| 41 | N | H | 18.2 |
| 42 | N | H | 11.2 |
| 46 | N | H | -23.2 |
| 49 | N | H | -39.3 |
| 50 | N | H | -41.9 |
| 53 | N | H | -35.8 |
| 54 | N | H | -29.3 |
| 55 | N | H | -30.4 |
| 56 | N | H | -21.0 |
| 57 | N | H | -21.3 |
| 58 | N | H | -24.5 |
| 60 | N | H | 15.1 |
| 63 | N | H | -27.7 |
| 65 | N | H | -15.0 |
| 67 | N | H | -32.2 |
| 70 | N | H | -26.3 |
| 73 | N | H | -24.8 |
| 75 | N | H | -21.0 |
| 76 | N | H | 14.0 |
| 78 | N | H | -19.3 |
| 82 | N | H | -14.5 |
| 86 | N | H | -17.5 |
| 88 | N | H | -29.1 |
| 90 | N | H | -0.1 |
| 91 | N | H | -35.4 |
| 94 | N | H | -27.6 |
| 95 | N | H | -37.1 |
| 98 | N | H | -29.3 |
| 99 | N | H | -17.5 |
| 105 | N | H | -19.8 |
| 110 | N | H | -34.2 |
| 113 | N | H | -32.5 |
| 128 | N | H | 1.5 |
| 137 | N | H | 6.7 |
| 138 | N | H | -4.9 |
| 139 | N | H | 19.1 |
| 140 | N | H | 22.8 |
| 141 | N | H | 19.9 |
| 144 | N | H | -13.5 |
| 145 | N | H | 17.1 |
| 146 | N | H | 6.1 |
| 150 | N | H | 10.9 |
| 151 | N | H | -9.0 |
| 156 | N | H | -24.9 |
| 157 | N | H | 23.7 |


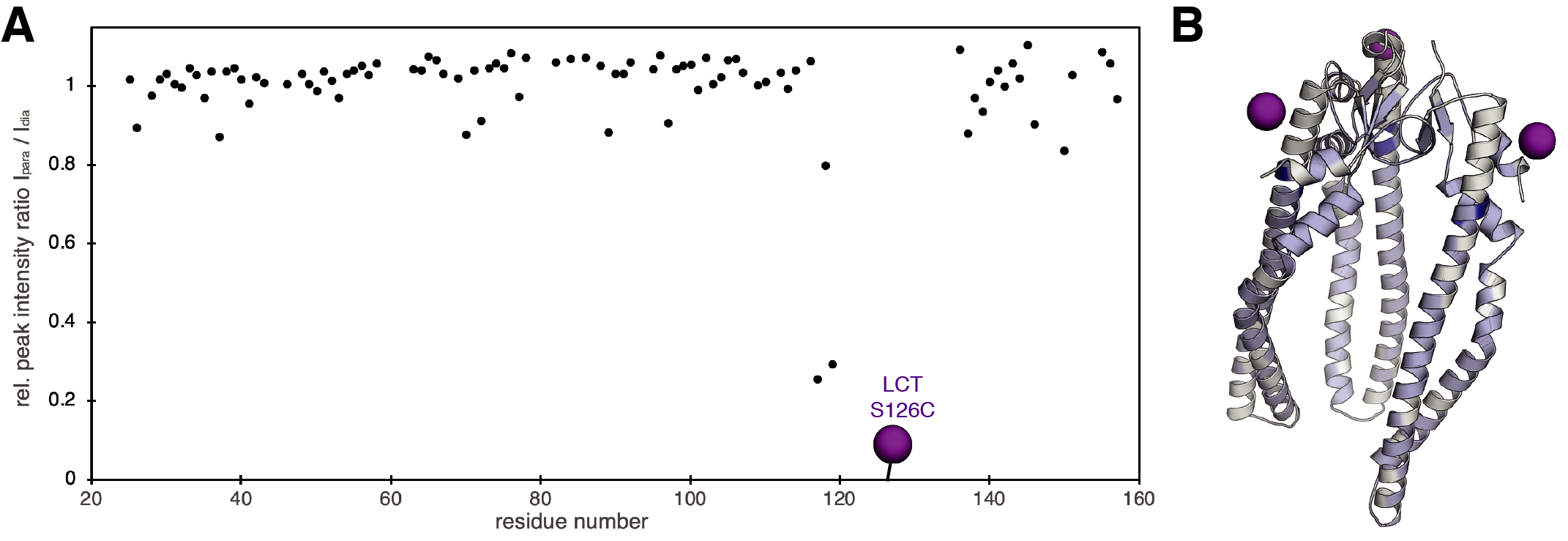


Figure S3. Paramagnetic relaxation enhancement (PRE) in Skp(S126C) tagged with Tm-M7PyThiazol-DOTA. (A) Ratio of peak intensities in the diamagnetic and paramagnetic spectrum. The purple sphere indicates the location of the LCT. (B) Visualization of the PRE on the Skp structure in ribbon representation. The peak intensity ratio is indicated by a color gradient from dark blue (I_para_ / I_dia_ = 0) to light blue (I_para_ / I_dia_ = 1). White residues are broadened beyond detection or unassigned.
